# Synthesis of Lithium Metal Oxide Nanoparticles by Induction Thermal Plasmas

**DOI:** 10.3390/nano6040060

**Published:** 2016-04-06

**Authors:** Manabu Tanaka, Takuya Kageyama, Hirotaka Sone, Shuhei Yoshida, Daisuke Okamoto, Takayuki Watanabe

**Affiliations:** 1 Department of Chemical Engineering, Faculty of Engineering, Kyushu University, 744 Motooka, Nishi-ku, Fukuoka 819-0395, Japan; mtanaka@chem-eng.kyushu-u.ac.jp; 2 Department of Chemical Systems and Engineering, Graduate School of Engineering, Kyushu University, 744 Motooka, Nishi-ku, Fukuoka 819-0395, Japan; kageyama.takuya.520@kyudai.jp (T.K.); h.sone@kyudai.jp (H.S.); daisuke.okamoto@kyudai.jp (D.O.); 3 Chemical Engineering Course, School of Engineering, Kyushu University, 744 Motooka, Nishi-ku, Fukuoka 819-0395, Japan; s.yoshida732@kyudai.jp

**Keywords:** thermal plasmas, lithium metal oxide, nanoparticle formation mechanism

## Abstract

Lithium metal oxide nanoparticles were synthesized by induction thermal plasma. Four different systems—Li–Mn, Li–Cr, Li–Co, and Li–Ni—were compared to understand formation mechanism of Li–Me oxide nanoparticles in thermal plasma process. Analyses of X-ray diffractometry and electron microscopy showed that Li–Me oxide nanoparticles were successfully synthesized in Li–Mn, Li–Cr, and Li–Co systems. Spinel structured LiMn_2_O_4_ with truncated octahedral shape was formed. Layer structured LiCrO_2_ or LiCoO_2_ nanoparticles with polyhedral shapes were also synthesized in Li–Cr or Li–Co systems. By contrast, Li–Ni oxide nanoparticles were not synthesized in the Li–Ni system. Nucleation temperatures of each metal in the considered system were evaluated. The relationship between the nucleation temperature and melting and boiling points suggests that the melting points of metal oxides have a strong influence on the formation of lithium metal oxide nanoparticles. A lower melting temperature leads to a longer reaction time, resulting in a higher fraction of the lithium metal oxide nanoparticles in the prepared nanoparticles.

## 1. Introduction

Nanoparticles have become widely utilized due to their enhanced and unique properties relative to bulk materials. The preparation of nanoparticles can be classified into physical and chemical methods. The physical methods include mechanical milling [1,2], laser ablation [3,4,5], and other aerosol processes with energy sources to provide a high temperature. Among these methods, attractive material processing with thermal plasmas have been proposed for the nanoparticles production. This is because thermal plasmas offer unique advantages; high enthalpy to enhance reaction kinetics, high chemical reactivity, rapid quenching rate in the range of 10^3–6^ K/s, and selectivity of atmosphere in accordance with the required chemical reactions. Thermal plasmas are capable of evaporating large amount of raw materials, even with high melting and boiling temperatures [6,7,8,9]. Furthermore, high-purity nanoparticles can be synthesized in an induction thermal plasma because thermal plasma can be generated in a plasma torch without internal electrodes [10,11]. These advantages of thermal plasmas have brought about advances in plasma chemistry and plasma processing [12,13,14,15].

Lithium metal oxides have attracted many researchers because of their unique properties as cathode for lithium-ion batteries [16,17,18], CO_2_ sorption material [19], and other magnetic, electrochemical materials. Layer structured LiCoO_2_ is widely employed as cathodes in commercial battery applications, in spite of its toxicity and high cost. To solve the economic and environmental problems, alternatives of LiCoO_2_ have been intensively explored. One of the alternatives is spinel-structured LiMn_2_O_4_, which provides a promising high-voltage cathode material for lithium-ion batteries owing to their high theoretical energy density, low cost, and eco-friendliness [18,20,21,22]. LiNiO_2_ is also a candidate due to its excellent cycle life, with negligible capacity fading *etc.* [17,23]. The liquid phase method is generally used in the synthesis of lithium metal oxide nanoparticles; however, productivity of the nanoparticles in the liquid phase method is insufficient for industrial application. Therefore, the synthesis method of lithium oxide nanoparticles with a high productivity is strongly demanded.

Thermal plasmas are expected to be promising energy sources to fabricate nanoparticles at high productivity from micron-sized powder as raw materials. Here, one-step synthesis of lithium-metal oxide nanoparticles with induction thermal plasma is studied. The purpose of the present study is to synthesize the lithium metal oxide nanoparticles via the induction thermal plasma and to investigate the formation mechanism of lithium-metal oxide nanoparticles. Different lithium metal systems—Li–Mn, Li–Cr, Li–Co, and Li–Ni—were compared to understand the formation mechanism.

## 2. Results and Discussion

### 2.1. Experimental Results

**Figure 1:**
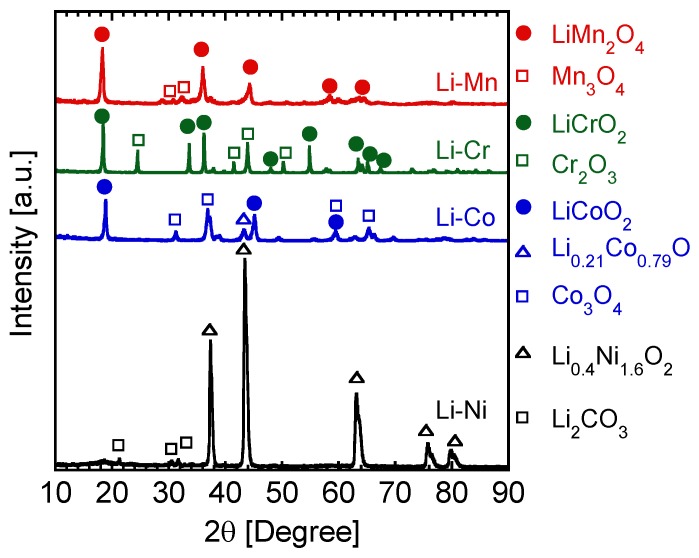
X-ray diffractometry (XRD) spectra of as-prepared nanoparticles by induction thermal plasma in different systems, Li–Mn, Li–Co, Li–Cr, and Li–Ni.

**Figure 2:**
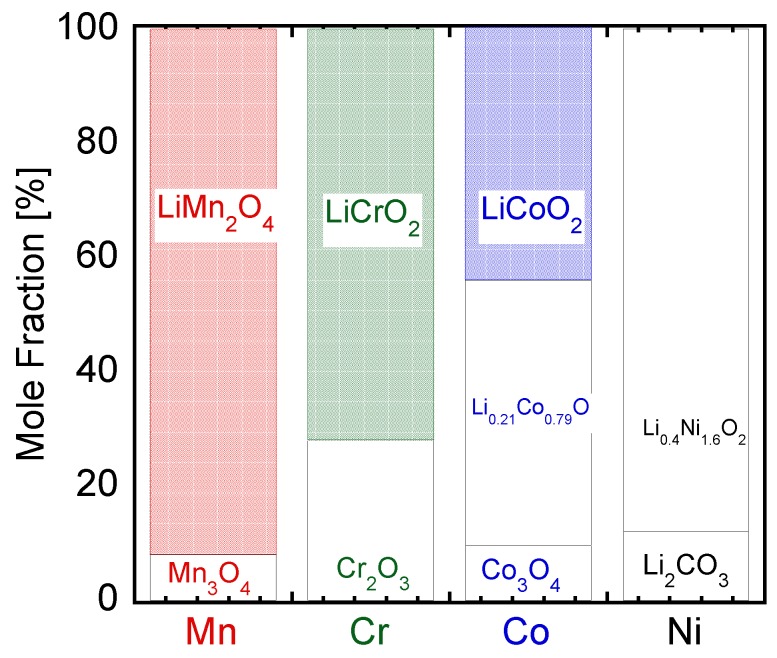
Composition of prepared nanoparticles by induction thermal plasma in different systems, Li–Mn, Li–Co, Li–Cr, and Li–Ni.

Figure 1 shows X-ray diffraction spectra of the prepared nanoparticles by the induction thermal plasma in different systems—Li–Mn, Li–Co, Li–Cr, and Li–Ni. In the case of the Li–Mn system, main diffraction peaks correspond to spinel-structured LiMn_2_O_4_, while diffraction peaks of Mn_3_O_4_ are also found. In cases of Li–Co and Li–Cr, layer-structured LiCoO_2_ and LiCrO_2_ were found as well as their oxides. In the Li–Ni case, Li_0.4_Ni_1.6_O_2_ and unreacted Li_2_CO_3_ were confirmed. These results clearly show the strong influence of the constituent metals on the formation of lithium oxide nanoparticles. Moreover, Figure 2 indicates composition of the prepared nanoparticles analyzed from X-ray diffractometry (XRD) spectra. These compositions of the prepared nanoparticles were used to evaluate the ratio of metal that reacted with Li. Evaluated reaction ratio will be discussed in the following section.

**Figure 3:**
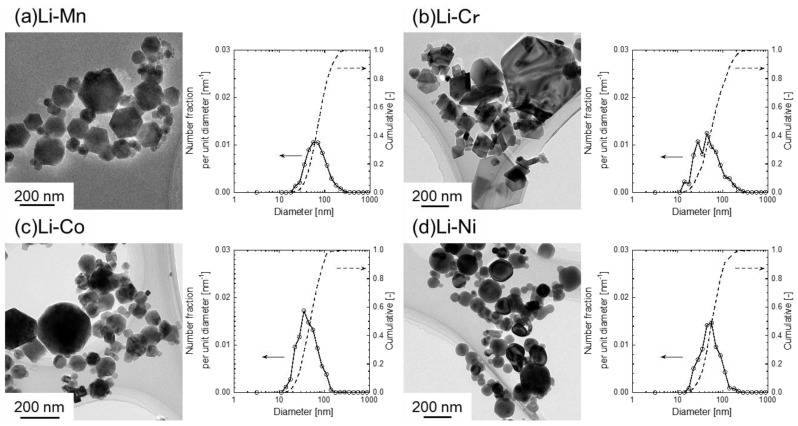
(**a**) Representative transmission electron microscopy (TEM) image and particle size distribution of prepared nanoparticles by induction thermal plasma in Li–Mn system; (**b**) that in Li–Cr system; (**c**) that in Li–Co system; (**d**) that in Li–Ni system.

Morphologies and particle sizes of the prepared nanoparticles were observed via transmission electron microscopy (TEM). Figure 3 shows the representative TEM images and the particle size distributions of the nanoparticles in different systems. Many spherical particles with 60 nm in mean diameter are observed in the Li–Ni system. In the cases of Li–Mn, Li–Co, and Li–Cr, most of the particles with 50–80 nm in mean diameters have polyhedral shapes including quadrangular, pentagonal, and hexagonal shapes. In particular, many particles with a hexagonal shape can be found as shown in Figure 3a. Hence, the spinel-structured LiMn_2_O_4_ is considered to have a hexagonal shape because LiMn_2_O_4_ is a major product in the Li–Mn system, according to XRD results.

**Figure 4:**
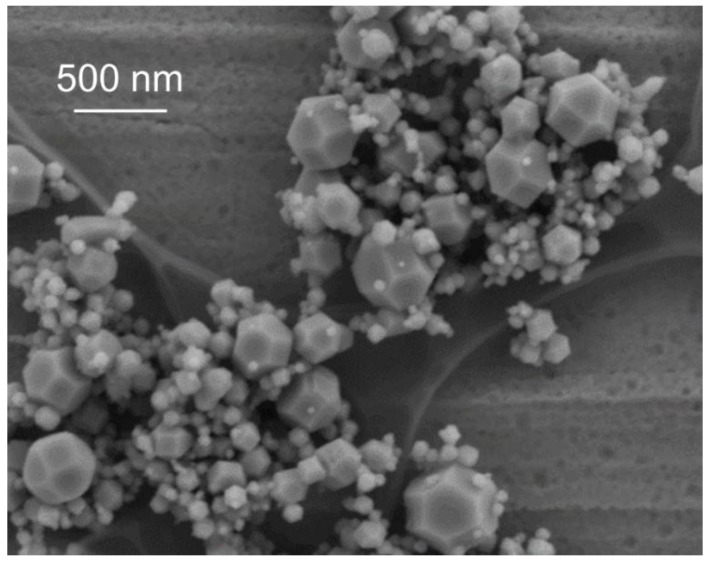
Representative scanning electron microscopy (SEM) image of prepared nanoparticles in Li–Mn system.

Scanning electron microscopy (SEM) observation was carried out to clarify morphology of LiMn_2_O_4_ more specifically than TEM observation. Figure 4 shows the representative SEM image of the prepared nanoparticles in the Li–Mn system. Many particles have truncated an octahedral shape as shown in Figure 4. Previous research on morphology of ferrite nanoparticles synthesized by the induction thermal plasma reported that the spinel-structured nanoparticles had a truncated octahedral shape [24]. The spinel-structured nanoparticles synthesized in thermal plasma have a truncated octahedral shape, although the stable structure of the spinel-structured particles are generally considered to be of an octahedral shape. The reason for this truncation is currently under investigation.

### 2.2. Nanoparticle Formation Mechanism

Homogeneous nucleation temperatures of metals considered in the present study were estimated based on nucleation theory considering non-dimensional surface tension [25]. The homogeneous nucleation rate *J* can be expressed as

(1) $$J=\frac{\beta_{ij}n_{s}^{2}S}{12}\sqrt{\frac{\Theta }{2\pi }}\text{exp}\left(\Theta -\frac{4\Theta^{3}}{27{\left(\text{ln}S\right)}^{2}}\right)$$

where *S* is the saturation ratio and *n*_s_ is the equilibrium saturation monomer concentration at temperature *T*. β is the collision frequency function. The dimensionless surface tension is given by the following equation:

(2) $$\Theta =\frac{\sigma s_{1}}{kT}$$

where σ is the surface tension and *s*_1_ is the monomer surface area. The surface tension and the saturation ratio have a dominant influence on determining the nucleation rate. Stable nuclei are observed experimentally when the nucleation rate is over 1.0 cm^−3^·s^−1^. Hence, the corresponding value of the saturation ratio is defined as the critical saturation ratio. The detailed procedure to estimate the nucleation rate and corresponding nucleation temperature can be found in previous works [10,25].

**Figure 5:**
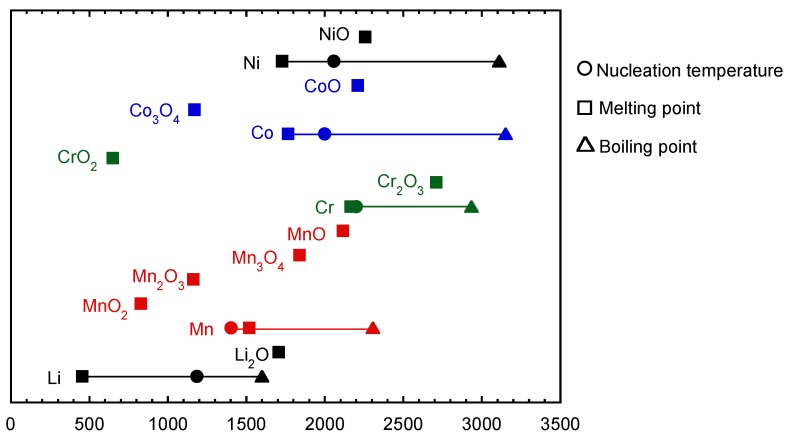
Relationship between nucleation temperature and melting and boiling points of considered metals and metal oxides.

The relationship between the calculated nucleation temperature and the boiling and melting points is summarized in Figure 5. Because of the unknown properties of metal oxides, only melting point oxides are plotted for metal oxides. These temperatures indicate that the melting points of the oxides are higher than the nucleation temperatures of pure metals in each Li–Me system. Therefore, nucleation of metal oxides is considered to occur at first. Li oxide and metal vapors co-condense onto the nuclei just after the nucleation starts.

The above mechanism can be proposed as a common mechanism for all considered Li–Me systems because the relationship between the nucleation temperature and the melting and boiling temperature of their oxides shows the same trend. However, experimental results show a different ratio of metal reactive with Li to total metal. In the cases of Li–Cr and Li–Mn, high ratios of the reactive metals were obtained, while a low ratio was obtained in Li–Ni system. Then, melting points of each oxide in different system were focused because the reaction rate of metal oxide particles and the condensed lithium would drastically decrease after complete solidification of the growing particles.

**Figure 6:**
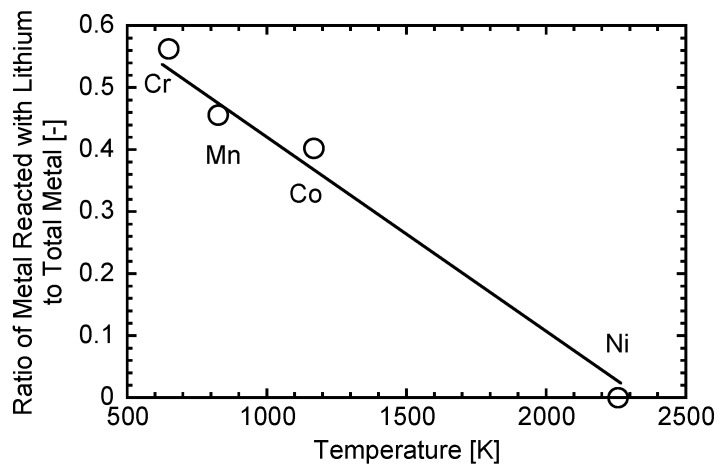
Relationship between lowest melting points of metal oxides and the reaction ratios for each Li–Me system.

**Figure 7:**
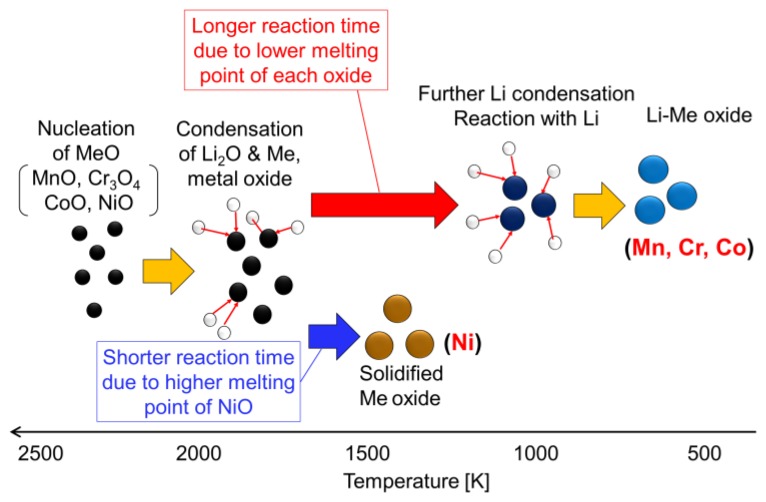
Conceptual diagram of formation mechanism of Li–Me oxide nanoparticles in induction thermal plasma.

Figure 6 shows the relationship between the lowest melting temperature of metal oxides for each Li–Me system and the reaction ratios. The results suggest a lower melting point of oxide leads to a higher reaction ratio. This can be explained by the different reaction time during the nanoparticle formation process. Figure 7 summarizes the formation mechanism of Li–Me oxide nanoparticles. A lower melting point of oxide leads to a longer residence time of growing particles in a liquid-like state, resulting in the longer reaction time with condensed lithium oxide. Consequently, a higher reaction ratio can be obtained in the Li–Me system, as in that of Li–Mn and Li–Cr. On the other hand, a shorter residence time of the growing particles in a liquid-like state leads to a shorter reaction time, resulting in a lower reaction ratio. These results suggest that the melting point of metal oxide has a strong influence on the reaction with lithium oxide.

The above mechanism can explain the obtained results well, although this is still only a hypothesis. Further experimental and theoretical investigation will be required to understand the formation mechanism of complicated oxide nanoparticles in thermal plasmas.

## 3. Experimental Section

### 3.1. Experimental Setup and Conditions

**Figure 8:**
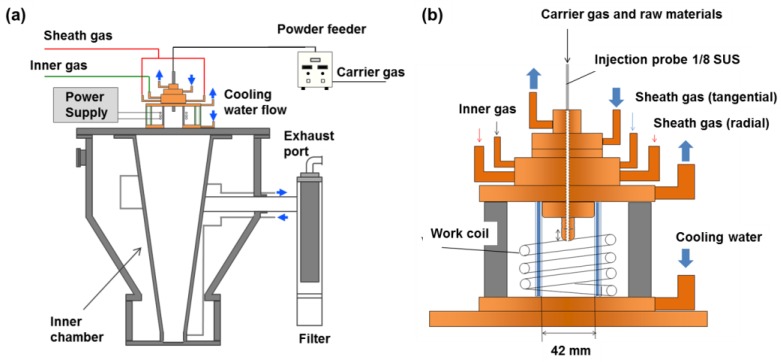
Schematic illustration of induction thermal plasma system for nanoparticles fabrication (**a**) and enlarged illustration of plasma torch (**b**).

A schematic illustration of the experimental setup for Li–Me oxide nanoparticle fabrication is presented in Figure 8a. This equipment is composed of the plasma torch, the reaction chamber where the nanoparticles are synthesized, and the filter unit. Figure 8b shows the enlarged illustration of the plasma torch. High temperature plasma of more than 10,000 K was generated in the plasma torch by induction heating at 4 MHz. The input power was controlled at 20 kW. Ar was used for the carrier gas of the raw powder at 3 L/min and inner gas at 5 L/min. A mixture of argon and oxygen were used for the plasma forming gas at 60 L/min. Mixture of Li_2_CO_3_ with 3.5 μm in diameter and metal or metal oxide with 3–10 μm in mean diameter were injected from the powder feeder by Ar carrier gas. Different metals including Mn, Cr, Co, and Ni were compared to investigate the formation mechanism of Li–Me (Mn, Cr, Co, and Ni) nanoparticles. The composition ratio of Li_2_CO_3_ to metal was 0.5. The powder feed rate was fixed at 400 mg/min. These experimental conditions are listed in Table 1.

Prepared nanoparticles were collected from the filter and the inner wall of the reaction chamber. Unevaporated raw materials were not confirmed according to SEM observation of the collected particles. This fact implies that the fed raw materials were completely evaporated in the high temperature region of the thermal plasma and converted into nanoparticles during the quenching process. Therefore, the mole fractions of the Li–Me oxides indicated in Figure 2 correspond to the yields of the Li–Me oxide in this nanoparticle fabrication process by the induction thermal plasma.

### 3.2. Characterization of Prepared Nanoparticles

The phase identification of the prepared nanoparticles was determined by X-ray diffractometry (XRD, Multiflex, Rigaku Co., Tokyo, Japan), operated with Cu Kα source (λ = 0.1541 nm). The diffraction data was collected using a continuous scan mode with a speed of 2 degree/min in the region of 10–90 degrees with a step size of 0.04 degrees. The accelerating voltage and applied current was 40 kV and 50 mA, respectively. The quantitative analysis of the composition of the prepared nanoparticles was conducted based on the whole-powder-pattern-decomposition (WPPD) method with the assumption that no amorphous particles were included in the prepared nanoparticles.

The particle morphology and size distribution of the prepared nanoparticles were observed by TEM (JEM-2100HCKM, JEOL Ltd., Tokyo, Japan), operated at an accelerating voltage of 200 kV. The TEM specimens were prepared by dispersing the as-prepared nanoparticles in ethanol and placing a few drops of the dispersion on a carbon-grid. Furthermore, the 3D particle morphology was observed by field emission (FE)-SEM (SII TES+ Zeiss ULTRA55, Carl Zeiss, Oberkochen, Germany).

## 4. Conclusions

Lithium metal oxide nanoparticles were synthesized in induction thermal plasma and formation mechanism was investigated. Obtained remarks are as follows:

(a) Lithium metal oxide nanoparticles were synthesized in different Li–Me (Mn, Cr, Co, and Ni) systems. In the case of Li–Mn, Li–Cr, and Li–Co, lithium-metal oxide nanoparticles were successfully synthesized, while Li–Ni oxides were not synthesized in the Li–Ni system.
(b) The spinel-structured LiMn_2_O_4_ with a truncated octahedral shape was synthesized in Li–Mn system, although the stable shape of the spinel structure was an octahedral shape.
(c) The relationship between nucleation temperature and boiling and melting points of the considered metals and their oxides suggests the following formation mechanism: Metal oxide starts to nucleate at first. Then, vapors of metal and lithium oxide co-condense on the metal nuclei with an oxidation reaction.
(d) Melting point of metal oxides is an important factor in determining the final product of the Li–Me composite. A lower melting point of metal oxide leads to a longer reaction time, resulting in higher yields of the Li–Me composite.
(e) Nanomaterial fabrication with induction thermal plasma enables the production of high-purity nanoparticles of Li–Me oxide at high productivity.

## Figures and Tables

**Table 1:** Typical experimental conditions.

Plasma Conditions				
Input power	20 kW			
Frequency	4 MHz			
Pressure	101.3 kPa			
Sheath gas	Ar: 57.5 L/min		O_2_: 2.5 L/min	
Inner gas	Ar: 5 L/min			
Carrier gas	Ar: 3 L/min			
Discharge time	5 min			
Feed rate	400 mg/min			
**Raw Materials**				
System	Li–Mn	Li–Cr	Li–Co	Li–Ni
Raw powders	Li_2_CO_3_, MnO_2_	Li_2_CO_3_, Cr	Li_2_CO_3_, Co	Li_2_CO_3_, Ni
Li/Me ratio	0.5			

## References

[1] Janot R., Guerard D. (2002). One-step synthesis of maghemite nanometric powders by ball-milling. J. Alloys Compd..

[2] Pithawalla Y.B., El Shall M.S., Deevi S.C. (2000). Synthesis and characterization of nanocrystalline iron aluminide particles. Intermetallics.

[3] Peterson S., Barickowski S. (2009). *In situ* bioconjugation: Single step approach to tailored nanoparticle-bioconjugates by ultrashort pulsed laser ablation. Adv. Funct. Mater..

[4] Mafune F., Khono J.Y., Takeda Y., Kondow T. (2001). Formation of gold nanoparticles by laser ablation in aqueous solution of surfactant. J. Phys. Chem. B.

[5] Yasukuni R., Horinaka T., Asahi T. (2010). Preparation of perylenediimide nanoparticle colloids by laser ablation in water and their optical properties. Jpn. J. Appl. Phys..

[6] Shigeta M., Watanabe T. (2005). Numerical analysis for co-condensation processes in silicide nanoparticle synthesis using induction thermal plasmas at atmospheric pressure conditions. J. Mater. Res..

[7] Shigeta M., Watanabe T. (2007). Growth mechanism of silicon-based functional nanoparticles fabricated by inductively coupled thermal plasmas. J. Phys. D.

[8] Shigeta M., Watanabe T. (2008). Numerical investigation of cooling effect on platinum nanoparticle formation in inductively coupled thermal plasmas. J. Appl. Phys..

[9] Shigeta M., Murphy A.B. (2011). Thermal plasmas for nanofabrication. J. Phys. D.

[10] Tanaka M., Noda J., Watanabe T., Matsuno J., Tsuchiyama A. (2014). Formation mechanism of metal embedded amorphous silicate nanoparticles by induction thermal plasmas. J. Phys. Conf. Ser..

[11] Cheng Y., Tanaka M., Watanabe T., Choi S.-Y., Shin M.-S., Lee K.-H. (2014). Synthesis of Ni_2_B nanoparticles by RF thermal plasma for fuel cell catalyst. J. Phys. Conf. Ser..

[12] Shigeta M., Watanabe T. (2015). Effect of precursor fraction on silicide nanopowder growth under a thermal plasma condition: A computational study. Powder Technol..

[13] Watanabe T., Liu Y., Tanaka M. (2014). Investigation of electrode phenomena in an innovative thermal plasma for glass melting. Plasma Chem. Plasma Proc..

[14] Liang F., Tanaka M., Watanabe T. (2014). Measurement of anode surface temperature in carbon nanomaterial production by arc discharge method. Mater. Res. Bull..

[15] Tanaka M., Watanabe T. (2013). Enhanced vaporization from molten metal surface by argon-hydrogen arc plasma. Jpn. J. Appl. Phys..

[16] Xiao X., Wang L., Wang D., He Q., Peng Q., Li Y. (2009). Hydrothermal synthesis of orthorhombic LiMnO_2_ nano-particles and LiMnO_2_ nanorods and comparison of their electrochemical performances. Nano. Res..

[17] Kalyani P., Kalaiselvi N. (2005). Various aspects of LiNiO_2_ chemistry: A review. Sci. Technol. Adv. Mater..

[18] Curtis C.J., Wang J., Schulz D.L. (2004). Preparation and characterization of LiMn_2_O_4_ spinel nanoparticles as cathode materials in secondary Li batteries. J. Electrochem. Soc..

[19] Ida J., Lin Y.S. (2003). Mechanism of high-temperature CO_2_ sorption on lithium zirconate. Environ. Sci. Technol..

[20] Shaju K.M., Bruce P.G. (2006). Macroporous Li(Ni_1/3_Co_1/3_Mn_1/3_)O_2_: A high power and high energy cathode for rechargeable lithium batteries. Adv. Mater..

[21] Canulescu S., Papadopoulou E.L., Anglos D., Lippert T., Schneider W., Wokaun A. (2009). Mechanism of the laser plume expansion during the ablation of LiMn_2_O_4_. J. Appl. Phys..

[22] Kim D.K., Muralidharan P., Lee H.-W., Ruffo R., Yang Y., Chan C.K., Peng H., Huggins R.A., Cui Y. (2008). Spinel LiMn_2_O_4_ nanorods as lithium ion battery cathodes. Nano Lett..

[23] Ohzuku T., Ueda A., Nagayama M. (1993). Electrochemistry and structural chemistry of LiNiO_2_ (R3m) for 4 volt secondary lithium cells. J. Electrochem. Soc..

[24] Swaminathan R., Willard M.A., McHenry M.E. (2006). Experimental observations and nucleation and growth theory of polyhedral magnetic ferrite nanoparticles synthesized using an RF plasma torch. Acta Mater..

[25] Girshick S.L., Chiu C.-P., McMurry P.H. (1990). Time-dependent aerosol models and homogenous nucleation rates. Aerosol Sci. Technol..

